# Histology-validated electromagnetic characterization of ex-vivo ovine lung tissue for microwave-based medical applications

**DOI:** 10.1038/s41598-024-55035-3

**Published:** 2024-03-11

**Authors:** Klementina Vidjak, Laura Farina, Ritihaas Surya Challapalli, Anne Marie Quinn, Martin O’Halloran, Aoife Lowery, Giuseppe Ruvio, Marta Cavagnaro

**Affiliations:** 1 Department of Information Engineering, Electronics, and Telecommunications, Sapienza University of Rome, Rome, Italy; 2 Endowave Ltd., Galway, Ireland; 3 Discipline of Surgery, Lambe Institute for Translational Research, University of Galway, Galway, Ireland; 4 Department of Anatomic Pathology, University Hospital Galway, Galway, Ireland; 5 Translational Medical Device Lab, National University of Ireland Galway, Galway, Ireland

**Keywords:** Biomedical engineering, Outcomes research, Translational research

## Abstract

Microwave thermal ablation is an established therapeutic technique for treating malignant tissue in various organs. Its success greatly depends on the knowledge of dielectric properties of the targeted tissue and on how they change during the treatment. Innovation in lung navigation has recently increased the clinical interest in the transbronchial microwave ablation treatment of lung cancer. However, lung tissue is not largely characterized, thus its dielectric properties investigation prior and post ablation is key. In this work, dielectric properties of ex-vivo ovine lung parenchyma untreated and ablated at 2.45 GHz were recorded in the 0.5–8 GHz frequency range. The measured dielectric properties were fitted to 2-pole Cole–Cole relaxation model and the obtained model parameters were compared. Based on observed changes in the model parameters, the physical changes of the tissue post-ablation were discussed and validated through histology analysis. Additionally, to investigate the link of achieved results with the rate of heating, another two sets of samples, originating from both ovine and porcine tissues, were heated with a microwave oven for different times and at different powers. Dielectric properties were measured in the same frequency range. It was found that lung tissue experiences a different behavior according to heating rates: its dielectric properties increase post-ablation while a decrease is found for low rates of heating. It is hypothesized, and validated by histology, that during ablation, although the tissue is losing water, the air cavities deform, lowering air content and increasing the resulting tissue properties.

## Introduction

In recent years, medical applications of electromagnetic fields are increasing, both with reference to diagnosis and therapy. Among therapies, microwave thermal ablation (MTA) utilizes electromagnetic fields at microwave frequencies to destroy malignant tissue^1^. This technique is considered minimally invasive as it uses an ablation antenna which is inserted directly into the tumor. The ablation antennas are designed so that they are matched to efficiently deliver energy at the desired frequency in the targeted tissue. For this reason, it is necessary to characterize the tissues targeted by MTA from the electromagnetic point of view. Besides MTA, another technique that benefits from the knowledge of dielectric properties is microwave imaging (MWI) which, based on the dielectric contrast, can determine the position of healthy and malignant tissue, and both diagnose the presence of tumors and enable monitoring of the MTA treatment^2–4^.

The use of MTA for the local treatment of pulmonary malignances is gaining more and more interest recently thanks to the advancement in bronchoscopy and lung robotic navigation enabling transbronchial MTA treatments^5–8^. Therefore, there is an urgency to thoroughly investigate the lung dielectric properties, pre-, peri- and post-ablation treatment and enable accurate application of microwave based clinical solution.

Many tissues are widely characterized in the microwave frequency range, including muscle^9,10^, heart^11^, and especially liver^10,12–14^. Lung tissue is one of the least characterized tissues due to its heterogeneity and difficulty to handle^15,16^. Furthermore, there are several works which have shown that lung dielectric properties are greatly influenced by the organ air content and its variation with the breathing cycle^17–19^.

Two studies have been conducted to investigate the temperature dependence of dielectric properties of lung tissue in the ablative temperature ranges^20,21^. Bonello et al. conducted experiments on 17 ex-vivo ovine lung tissue in the 0.5–8 GHz frequency range. They used both an MTA antenna and a microwave oven (MWO) to heat the samples. In case of MTA, the samples were treated for 3 min with an antenna input power of 80 W, and the dielectric properties were measured every 15 s with the open-ended probe placed perpendicular to the antenna inside the tissue. During MWO heating, the samples were heated for 45 s at 120 W to achieve a 5 °C temperature increase. Afterwards, the dielectric properties were measured three times at the same spot on the tissue and the sample was returned to the oven for another 45 s. This procedure was repeated until the tissue reached ~70 °C, when it was no longer possible to keep the temperature constant after the MWO heating^21^. They observed a decrease in dielectric properties with temperature with both heating modalities. Sebek et al. measured the properties of 30 ex-vivo ovine and porcine samples in the 0.5–6 GHz frequency range for temperatures between 31 and 150 °C^20^. The samples were placed in a custom-made copper box heated using a hot plate^20^. Again, the properties decreased with temperature. These findings suggest that the lung tissue behaves similarly to other tissues, where the dielectric properties decrease at ablative temperatures due to the evaporation of water^12^.

However, the dielectric properties of lung tissue post-ablation, after a typical treatment duration (i.e., about 10 min^22^) and once it is cooled back to room temperature are not reported in literature, nor is the rate at which the temperature of the tissue changed during the study. These gaps are addressed in this work.

In this study, both the MTA and MWO approaches were used to investigate the changes in the dielectric properties of lung tissue subject to microwave heating. In total, 13 ex-vivo ovine lungs were ablated for 10 min with a power of 60 ± 1 W supplied at the antenna. The dielectric properties of non-ablated and ablated tissue were measured in the 0.5–8 GHz frequency range. The measurements of these two types of tissue were analyzed and fitted to 2-pole Cole–Cole relaxation models^23^. Based on the parameter changes observed between the relaxation models and their association to different dispersion mechanisms, the physical changes occurring in the lung tissue post-ablation were discussed alongside with histological observation. Additionally, MWO heating was applied to verify the dielectric properties measured after ablation. For this purpose, one porcine sample was used to recreate the experimental setup used by Bonello et al^21^, 5 ex-vivo porcine lung tissue samples were heated for 5 min at the power of 120 W to recreate the energy deposition of MTA, and four ex-vivo ovine lung tissue samples were MWO heated twice for 45 s at the power of 800 W. The MWO measurements were conducted independently in two different labs (Translational Medical Device laboratory (TMDLab) in Galway Ireland on ovine lung, and Microwave Lab, Department of Information Engineering, Electronics and Telecommunications (DIET) at Sapienza University of Rome, in Italy on porcine lung). In the following, the methods are described first, then results follow.

## Methods

### Lung tissue preparation

All ex-vivo ovine lung samples used in the experiments at the Translational Medical Device laboratory (TMDLab) in Galway Ireland, were obtained freshly from a local abattoir and used the same day. Porcine samples used in the experiment at the Department of Information Engineering, Electronics and Telecommunications (DIET) at Sapienza University of Rome, in Italy, were obtained ~1 day after euthanasia. All samples were handled and stored to minimize tissue dehydration and deterioration^23^. In total, 13 ex-vivo ovine lung samples and 6 ex-vivo porcine lung samples were used for this study. The ovine samples were taken from 11 different animals, while the porcine samples all originated from the same animal.

The samples were used either in the state in which they were received from the abattoir, i.e. collapsed (in the following cited as “deflated state”), or in the inflated state^19^. It is worth noting that the collapsed lung air is physiologically still present due to the alveolar structure of the tissue, so that it cannot be compared directly with the deflated lung as reported in other literature reports^24^. To achieve the inflated state, the sample was artificially/mechanically inflated. The pleura had to be intact, without any obvious ruptures that could prevent the inflation process and with the heart in place to guarantee the integrity of the pulmonary circulation, thus minimizing blood loss/sample exsanguination. Prior to inflation, the trachea was cleaned and prepared to be connected with the inflation tube, removing the esophagus, if present. The sample was then connected through the inflation tube to the inflation setup which consisted of a mini 12V DC air pump, a DC power supply (TENMA 72-8695A) for the pump, pressure gauge (Testo 510i) and tubing^19^.

To fully inflate the lung, the sample was fed with air at the constant pressure of around 29 mbar during the ablation procedure as well as for 2 h after completion of the ablation procedure. The pressure level chosen enables lung tissue recovery from atelectasis occurring after the organ removal from the body^25^. Inflated samples were used for the MTA experiments.

### Lung tissue ablation

**Figure 1:**
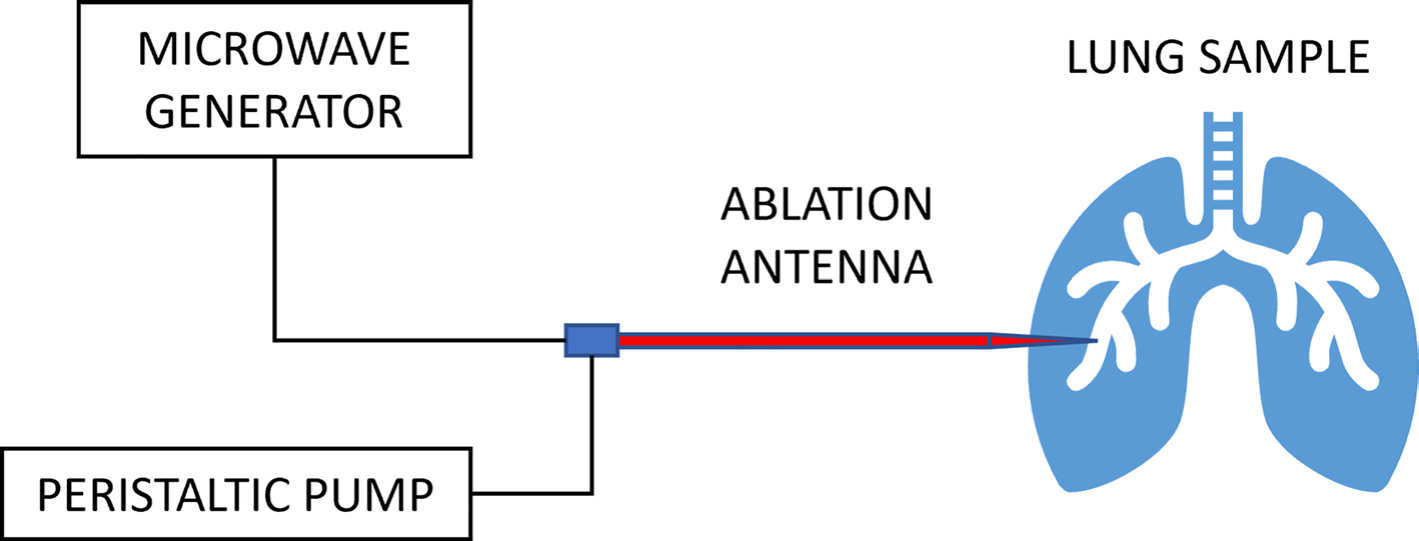
Sketch of the ablation setup.

The ablation setup in the TMD Lab is sketched in Fig. 1. The setup included a dipole ablation antenna, inserted percutaneously into the lung parenchyma ensuring 6 cm insertion depth; a microwave generator (Sairem Microwave Generator 2000 W at 2450 MHz); a cooling system with 1L water reservoir (at 18–19 °C), and a peristaltic pump with tubing (Fisherbrand™ DP2000 Dispensing Pump System). An infrared thermometer (Fluke 62 MAX Mini Infrared Thermometer) was used for measuring surface tissue temperature.

N = 13 ablations were performed at 2.45 GHz, for 10 min, supplying $60 \pm 1$ W at the antenna. During the procedure, reflected power was monitored. Upon ablation completion, the antenna was replaced by a q-tip rod to mark the antenna position and prevent air leakages. The lungs were covered in cling film to minimize dehydration. Afterwards, the sample was left to cool for 2 h at room temperature with constant air supply (in case of inflated tissue). After the cooling process, the sample was cut along the insertion axis of the antenna marked with the q-tip so that a section of the sample over the central plane of the ablation was obtained. Once the tissue was cut, two sections of the sample were obtained: the bottom and the upper half of the ablated tissue. The bottom half of the ablated tissue was used for dielectric properties measurement, while the matching top half was stained with triphenyltetrazolium chloride (TTC) to mark the ablation zone^26^. TTC staining was used to determine the thermal ablation zone margin, often poorly visible in lung tissue. TTC stains red when bonds with the cells mitochondrial activity, that is lost in ablated tissue^26^. Two zones can be identified: the TTC negative ablated zone and the TTC diminished ablation zone. TTC negative area is not stained from TTC due to complete absence of mitochondrial activity; TTC diminished zone is defined as a transition zone between ablated and non-ablated area where there may be still some mitochondrial activity^26^. A digital caliper was used to measure the length and diameter of the stained ablation zone.

### Microwave oven heating of lung tissue

Experiments with ovine tissue were performed in the TMD Lab; while experiments with porcine tissue were performed at DIET.

All samples used for MWO heating were in a deflated state. Six samples were excised from one ex-vivo porcine lung tissue (@DIET, Rome, Italy). Among these samples, one sample was used to re-create Bonello et al experimental setup where the sample was heated several times for 45 s at 120 W to achieve a 5 °C temperature increase^21^. However, a 45 s session was insufficient for achieving 5 °C temperature increase in this experiment. Therefore, after six unsuccessful attempts of achieving exactly 5 °C increase, during the seventh and eighth session, the sample was left in the oven for 90 and 180 s respectively (temperature was checked each 45 s to see how much it increased). The remaining five samples were MWO heated for 5 min at 120 W to match the energy deployed into the tissue during the 10 min of ablation at 60 W. Four pieces were cut-out from four ex-vivo ovine lung tissue samples (@ TMD Lab, Galway, Ireland) and heated twice at the power of 800 W, each time for 45 s. The overview of the described experiments is given in Table 1.

**Table 1:** Overview of MWO heating experiments.

Tissue type	No. of samples	Heating power (W)	Cumulative heating duration (s)	Heating energy (kJ)^a^
Ex-vivo porcine	1	120	45 s	5.4
			90 s	10.8
			135 s	16.2
			180 s	21.6
			225 s	27.0
			270 s	32.4
			360 s (90 s in the oven)	43.2
			540 (180 s in the oven)	64.8
	5	120	5 min	36
Ex-vivo ovine	4	800	45 s	36
			90 s	72

^a^Reference energy for 60W 10 min ablation is 36 kJ.

The energy deployed into the tissue during the 10 min of ablation at 60 W is the same as during the 5 min at 120 W in the MWO heating, and first 45 s of MWO heating at 800 W. It is worth noticing here that in the replication of the Bonello et al study^21^ the energy transferred to the tissue in each heating session is 15% of the reference MTA one.

### Dielectric properties measurements

The measurements of the dielectric properties were performed using the open-ended coaxial probe technique^27,28^. The measurement setup used at the TMD Lab for measurements of ovine tissue consisted of a slim-form probe (Keysight 85070E, Santa Rosa, CA, USA) connected to a VNA (Keysight E5063A Vector Network Analyzer, Santa Rosa, CA, USA) and the Keysight software for dielectric properties de-embedding (N1500A-004 Material Measurement Suite, Santa Rosa, CA, USA). The measurement frequency range was 0.5–8 GHz with 151 linearly spaced points. The measurement setup used at DIET for measurements of porcine tissue included a slim-form probe (Keysight 85070E, Santa Rosa, CA, USA), connected to a VNA (Agilent E8363C PNA Vector Network Analyzer) device and an in-house de-embedding software based on the Stuchly and Stuchly model^29^. The measurement frequency range for these experiments was 0.5–8 GHz with 151 linearly spaced points. In this setup, a fiber optic probe (PRB-G40-2M-STM-MRI probe, FTX-300-LUX+, 3 Channel Thermometer, Osensa Innovations, Burnaby, BC, Canada) was joined to the slim form probe so as to put the tips of the two probes at the same height. This arrangement allowed simultaneous measurements of the temperature during dielectric properties measurements.

In all cases, a lift was used to place the measured sample in contact with the probe to minimize the movements of the probe and coaxial cable connecting it to the VNA. Additionally, the lift was used to adjust the pressure to ensure good probe-sample surface contact while avoiding puncturing the tissue. The calibration was performed with air (open circuit), short circuit and distilled water. The Keysight software ensures the accuracy of 5 % when calibration is performed properly^28^. To verify the calibration, 0.1 mol natrium chloride (NaCl) solution was measured^30^.

### Microwave ablated samples

**Figure 2:**
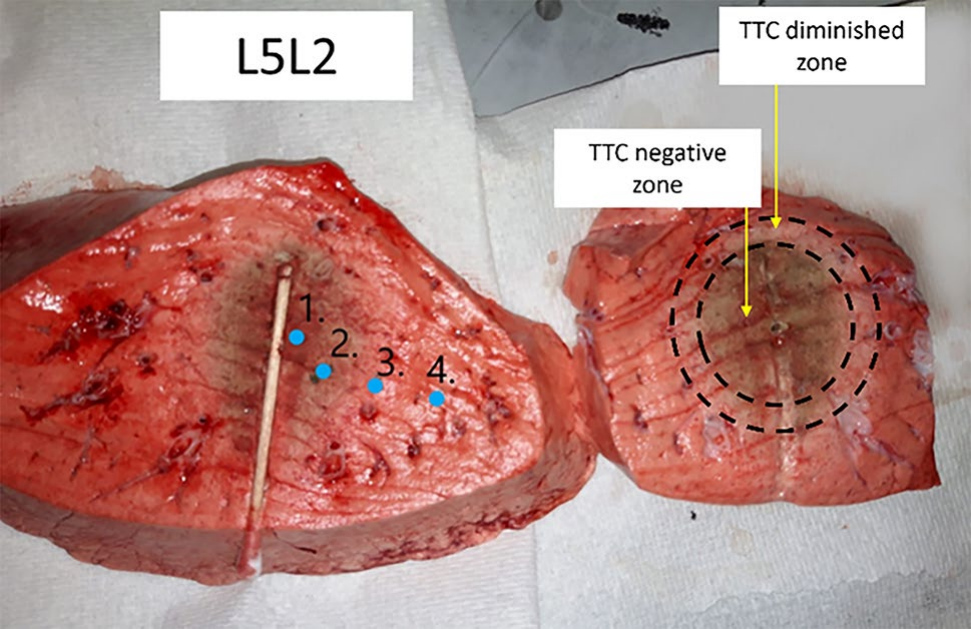
Comparison between the portion of the tissue used for dielectric measurements (left) and for TTC staining (right).

The dielectric properties of samples used for MWA experiments in the TMD Lab were measured in both non-ablated and ablated area of the tissue (i.e. on areas identified on the bottom half of the sample post-ablation). As stated previously, these measurements were performed once the tissue had cooled down back to the room temperature. On average, the tissue temperature during these measurements was 24.7 ± 1.3 °C. Measurements were repeated five consecutive times for each measurement point. The probe was always removed from the tissue and cleaned with alcohol wipes in between consecutive measurements. Once the measurement sequence was complete, the measured location was marked for later comparison with the matching TTC stained portion of the sample. An example of comparison between the portion of the tissue used for dielectric measurements and the portion used for TTC staining can be seen in Fig. 2. The comparison between the two portions of the sample (i.e. upper and bottom half of the sample) was used to classify the dielectric measurement location as non-ablated or ablated. In total 20 measurement locations were classified as non-ablated and 36 as ablated, across all samples.

The dielectric properties of each measurement location were represented as the average of the five measurement repetitions taken on the same spot. Afterwards, the dielectric properties measured across all locations in either ablated or non-ablated tissue were averaged to obtain the dielectric properties of the two tissue classes.

### Microwave oven heated samples

The dielectric properties of samples used for MWO heating were measured before and after MWO heating.

The dielectric properties of the one porcine sample used to re-create the experimental procedure from literature^21^ were measured after each MWO session of either 45 or 90 s, depending if the temperature increment of 5 °C was achieved or not. The measurements after each session were always taken on the same spot of the tissue and repeated 3–5 times depending on how stable the tissue temperature was.

The dielectric properties of five porcine samples heated for 5 min at 120 W were measured right after the MWO heating and after ~5 min once the tissue temperature dropped below 40 °C. Both measurements were repeated five times on the same location and averaged. These measurements were classified in two categories, as the measurements “post-MWO heating” and “post-MWO heating and cooling” respectively.

As previously reported, in these measurements, temperature was controlled and recorded by a fiber optic probe (Osensa Innovations, Burnaby, BC, Canada) joined with the slim form probe.

For the four ovine samples, the dielectric properties were taken after each MWO session of 45 s at 800 W and repeated five times at three different measurement spots. Afterwards the measurements were averaged across the five repetitions and measurement locations. These measurements were classified in two categories, as the measurements after 45 and 90 s respectively. In this case, the temperature of the sample was measured on the surface of the sample using an infrared gun (N85FR, InfraRed Thermometer, Precision Gold, Lafayette, CO, USA). After both 45 and 90 s MWO sessions, the temperature was dropping very fast (i.e. the initial measurement point had a temperature 50–60 °C while the last measurement point had a temperature of about 25–30 °C).

### Histology analysis

After the dielectric measurement procedure was completed, seven samples of tissue used in MWA experiments in TMD Lab (1 cm to 2 cm wide and less than 5 mm thick) were taken from three lung samples (2 inflated, 1 not inflated) and embedded in formalin; three samples were taken from inside the ablation zone; three from not ablated tissue, and one included both ablated and not ablated tissue. For each sample it was noted the parent lung sample, if it was subject to inflation and ablation. Samples were embedded in formalin 2 to 4 h after ablation end.

The tissues were transferred into 4% paraformaldehyde (PFA) in PBS (pH.6.9) and stored at room temperature for at least 24 h until processing and embedding^31^. The tissues were then processed in series of alcohols and embedded in Paraffin using Excelsior ES tissue processor (A82310100, Thermo Scientific, Waltham, MA, USA) and HistoCore Arcadia H embedding system (14039357258, Leica Biosystems, Wetziar, Germany).

The paraffin-embedded blocks were sectioned to obtain 5 µm sections and stained with haematoxylin and eosin (H&E), dehydrated, and mounted with dibutylphthalate polystyrene xylene (DPX). Images of the slides from H&E were acquired using Olympus Virtual Slide Scanner VS120-L100-W and OlyVia v3.3 (Olympus Life Science Waltham, MA, USA).

### Dielectric properties data analysis

#### Uncertainty analysis

The two main contributions to the uncertainty of the measure are the accuracy of the measurement (Type B uncertainty) and repeatability of the measurement (Type A uncertainty)^32^. The first contribution was considered equal to the percentage deviation between measured and literature data of 0.1 M NaCl solution^30,33^, and the latter contribution were random errors obtained from the repeated measurements at the same spot on the lung surface and calculated as SDM (percentage standard deviation of the mean)^32,33^. The number of repetitions varied from 3 to 5, depending on the experimental setup described in the section “Dielectric properties measurements”.

The accuracy component of uncertainty was calculated using the following equation:

1 $$\begin{array}{c}{ACC}_{x}=\frac{\left|{x}_{ref}-{x}_{meas}\right|}{{x}_{ref}}*100, \end{array}$$

where: ${x}_{ref}$ is either the reference permittivity or equivalent conductivity of the 0.1 M NaCl solution, obtained from the literature^30^, ${x}_{meas}$ is either the measured permittivity or equivalent conductivity of the 0.1 M NaCl solution.

The SDM component was calculated as^32^:

2 $$\begin{array}{c}SDM=\sqrt{\frac{\sum_{i=1}^{n}{\left({x}_{i}-\overline{x }\right)}^{2}}{n\left(n-1\right)}}, \end{array}$$

where ${x}_{i}$ is the ${i}$ th data point, $\overline{x }$ is the average value obtained from the total $n$ measurements.

SDM is expressed in percentage with:

3 $$\begin{array}{c}SDM\left[\mathrm{\%}\right]=\frac{SDM}{\overline{x} }*100, \end{array}$$

The uncertainty components related to the systematic errors (accuracy) can be modelled by a rectangular (R) distribution and uncertainty component related to the random errors (repeatability) can be assumed as normally (N) distributed^32^. Therefore, the uncertainty component attributed to accuracy is:

4 $$\begin{array}{c}{u}_{acc.}=\frac{{a}_{acc}}{{b}_{acc}}=\frac{{ACC}_{{\varepsilon }^{\mathrm{^{\prime}}}/\sigma }[\mathrm{\%}]}{\sqrt{3}}. \end{array}$$

And the uncertainty component attributed to repeatability is:

5 $$\begin{array}{c}{u}_{rep.}=\frac{{a}_{rep}}{{b}_{rep}}=\frac{SDM \left[\mathrm{\%}\right]}{1}. \end{array}$$

Finally, combined uncertainty is calculated as the square root of the arithmetic sum of the square of the best estimation of each uncertainty ${u}_{i}$ (${u}_{rep.}$ and ${u}_{acc.}$):

6 $$\begin{array}{c}{u}_{c}=\sqrt{\sum_{i=1}^{n}{u}_{i}^{2}}. \end{array}$$

#### Relative change of dielectric properties

Measurements of each sample were analyzed separately. In the case of MTA heating, a comparison between the measured dielectric properties of non-ablated and ablated tissue of each sample was performed. Non-ablated tissue measurements of each sample were averaged to represent the non-ablated properties of that particular sample, while the ablated measurement points were considered separately. Each ablated measurement point was compared to the non-ablated properties of the same sample to better correlate the changes introduced by the ablation procedure.

The relationship between non-ablated and ablated dielectric properties, permittivity and conductivity, was expressed as a relative change:

7 $$\begin{array}{c}\Delta x=\frac{{x}_{abl}-{x}_{non-abl}}{{x}_{non-abl}}*100, \end{array}$$

where ${x}_{abl}$ is either the permittivity or equivalent conductivity of ablated tissue and ${x}_{non-abl}$ is either permittivity or equivalent conductivity of non-ablated tissue.

The relative change results in either an increase or decrease in dielectric properties of ablated tissue with respect to the properties of non-ablated tissue.

#### 2-pole Cole & Cole model fitting

The dielectric properties of biological tissues are generally described in the literature with multi-pole Cole–Cole relaxation models^23,24^. In a recent study on dielectric properties of healthy lung tissue^19^, it was found that the 2-pole Cole–Cole model was sufficient for describing the dielectric properties of lung in the frequency range 0.5–8 GHz:

8 $$\begin{array}{c}\widehat{\varepsilon }\left(\omega \right)={\varepsilon }_{\infty }+\frac{{\Delta }_{1}}{1+{\left(j\omega {\tau }_{1}\right)}^{1-{\alpha }_{1}}}+\frac{{\Delta }_{2}}{1+{\left(j\omega {\tau }_{2}\right)}^{1-{\alpha }_{2}}}+\frac{{\sigma }_{s}}{j\omega {\varepsilon }_{0}}, \end{array}$$

where ε_∞_ is permittivity at infinite frequency, Δ_i_ is the change of permittivity of the ith dispersion, τ_i_ is the time constant of the ith dispersion, α_i_ is an empirical parameter for broadening the dispersion, σ_s_ is the static conductivity term, ω is the angular frequency, ε_0_ is the permittivity of vacuum. The index i = 1 represents the γ dispersion which is associated to the relaxation of water, and the index i = 2 represents the β dispersion, associated to passive cell membrane capacitance, intracellular organelle membranes and protein molecule response^34^.

The measured properties were fitted to the 2-pole Cole–Cole model given with Eq. (8) using the weighted least squares algorithm (W-LSM)^35,36^. This algorithm uses a complex weight factor $\dot{{w}_{i}}$ to control and improve the fitting. The non-linear least square method (LSM) was used with the Newton iterative method to minimize the total weighted and the squared error as:

9 $$\begin{array}{c}{E}^{2}=\sum_{i=1}^{{N}_{f}}\left[\frac{{\left\{{c}_{r}\left({\omega }_{i}\right)-{d}_{r}\left({\omega }_{i}\right)\right\}}^{2}}{{\left\{{e}_{r}\left({\omega }_{i}\right)\right\}}^{2}}+\frac{{\left\{{c}_{i}\left({\omega }_{i}\right)-{d}_{i}\left({\omega }_{i}\right)\right\}}^{2}}{{\left\{{e}_{i}\left({\omega }_{i}\right)\right\}}^{2}}\right], \end{array}$$

where: ${N}_{f}$ is the number of frequency points, ${c}_{r}\left({\omega }_{i}\right)$ and ${c}_{i}\left({\omega }_{i}\right)$ are the real and imaginary parts of the calculated permittivity (obtained through fitting), ${d}_{r}\left({\omega }_{i}\right)$ and ${d}_{i}\left({\omega }_{i}\right)$ are the real and imaginary parts of the measured permittivity, ${e}_{r}\left({\omega }_{i}\right)$ and ${e}_{i}\left({\omega }_{i}\right)$ are the real and imaginary parts of the allowable error $\dot{{e}_{i}}$.

The error $\dot{{e}_{i}}$ is chosen dependent on the permittivity^36^:

10 $$\begin{array}{c}\dot{{e}_{i}}\approx {\left\{\widehat{\varepsilon }\left({\omega }_{i}\right)\right\}}^{\xi }, \end{array}$$

where: $\widehat{\varepsilon }\left({\omega }_{i}\right)$ is the measured permittivity at frequency ${\omega }_{i}$, $\xi$ is a power factor, in this case equal to 0.75, so that more weight is put on the lower frequency data.

The success of this algorithm depends on the initial and boundary values chosen for the parameters. The boundary values used in this work were chosen based on the observations of the parameter values obtained by Gabriel et al. for the 4-pole Cole–Cole model of the inflated and deflated lung^24,37^; during the fitting they were then modified based on trial and error to achieve the lowest possible fitting error.

The fitting error was determined as an average across all frequencies for both the real and imaginary part of complex permittivity using the following expression:

11 $$\begin{array}{c}{Error}_{{\varepsilon }_{avg}^{\prime},{\varepsilon }_{avg}^{{\prime}{\prime}}}=\sum_{i=1}^{{N}_{f}}\frac{\left[\frac{{c}_{r,i}\left({\omega }_{i}\right)-{d}_{r,i}\left({\omega }_{i}\right)}{{d}_{r,i}\left({\omega }_{i}\right)}\right]*100}{{N}_{f}}. \end{array}$$

## Results

### Comparison of dielectric properties between non-ablated and ablated tissue

First, the dielectric properties measured for each ablated point on a specific sample were compared to the averaged dielectric properties of non-ablated tissue of the same sample. The dielectric property relative change post-ablation was calculated using Eq. (7). It was found that 86.1 % (31 out of 36) of the ablated tissue measured points exhibited an increase in dielectric properties compared to non-ablated tissue. The increase ranged from ~10% up to ~150%. The remaining data (13.9 %, corresponding to 5 points out of 36) did not exhibit an increase in dielectric properties; the properties either decreased (in 2 cases) or remained within the experimental uncertainty of the non-ablated measurement (in 3 cases). Relative changes were not constant across the considered frequency range. At lower frequencies, until ~2 GHz, the relative change in permittivity decreases as frequency increases; from 2 GHz the relative change in permittivity is constant. A more complex behavior is observed for conductivity. Based on the obtained results, three patterns of the general behavior of the relative change in the conductivity across the frequency range were observed:

- Group 1—The relative change increased with increasing frequency (8 points, 22.2%),
- Group 2—The relative change decreased with increasing frequency (21 points, 58.3%),
- Group 3—The relative change was about constant across the entire frequency range (7 points, 19.4%).

**Figure 3:**
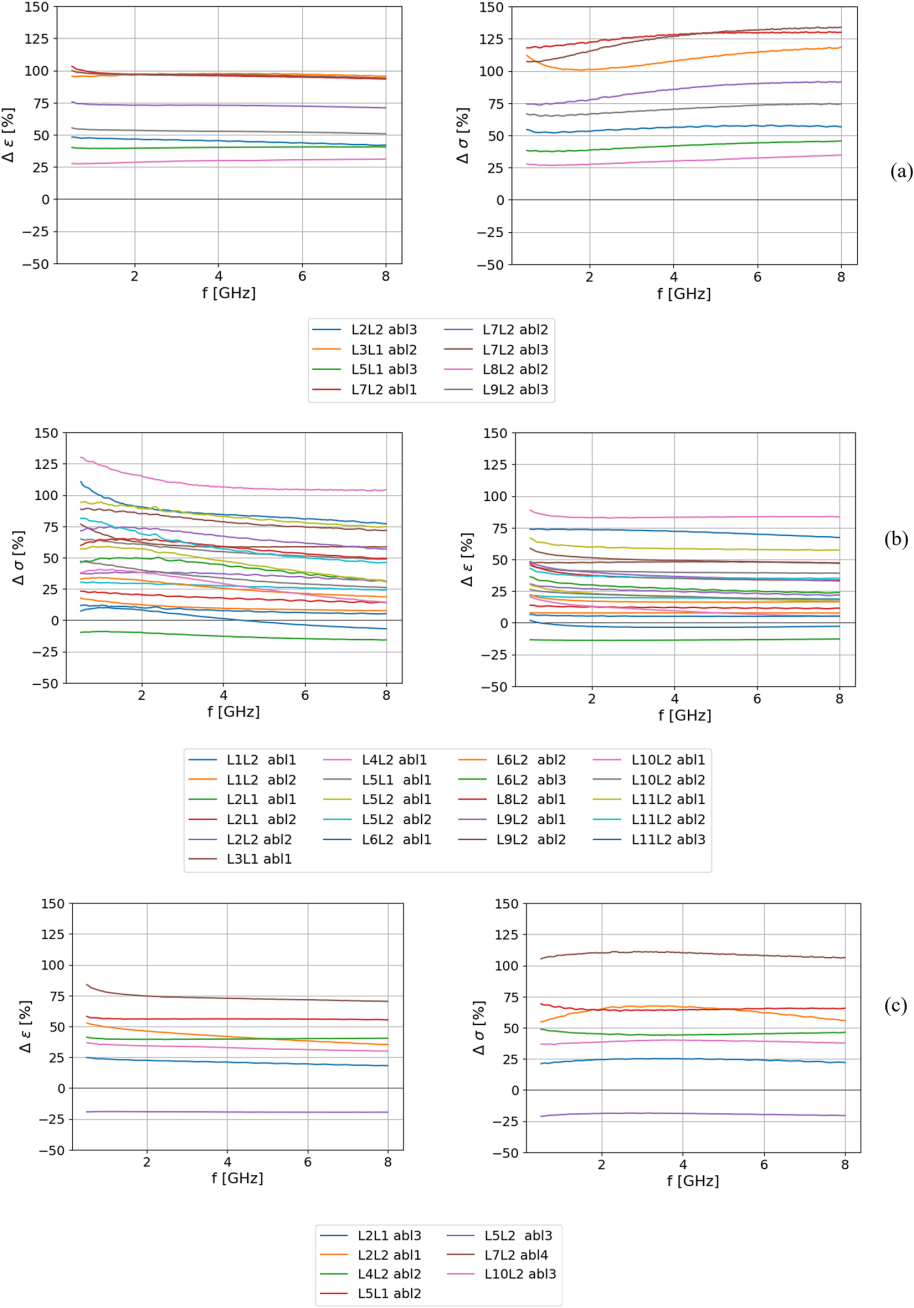
Relative change of dielectric properties of points in the ablated area: (**a**) Group 1, (**b**) Group 2 and (**c**) Group 3.

The relative percentage changes of permittivity and conductivity calculated for all measurement points are presented in Fig. 3. The abbreviations “LxLy” in the legend of Fig. 3 are lung sample labels, where “x” represents the parent animal number, and “y” indicates the left (y = 1) or right lung (y = 2). Furthermore, abbreviation “abl” stands for measurement points in the ablated area taken on the sample followed by the progressive measurement number. The dominant pattern is the one depicted for Group 2 (panel b) in Fig. 3). These measurement points were all taken in the ablated area close to the axis of the ablation antenna ($\le 1$ cm; within 50% of the total ablation extension: ‘abl1’ and ‘abl2’ labels dominate), i.e. in the TTC negative area. Points taken in the outer regions of the ablation zone (‘abl3’, ‘abl4’ and ‘abl5’), i.e. in the TTC diminished area, show different relative changes across the frequency range (1st, and 3rd groups). In total, 17 points in Group 2 were taken directly in the TTC negative area, the remaining 4 points in the group were taken in the TTC diminished area.

**Figure 4:**
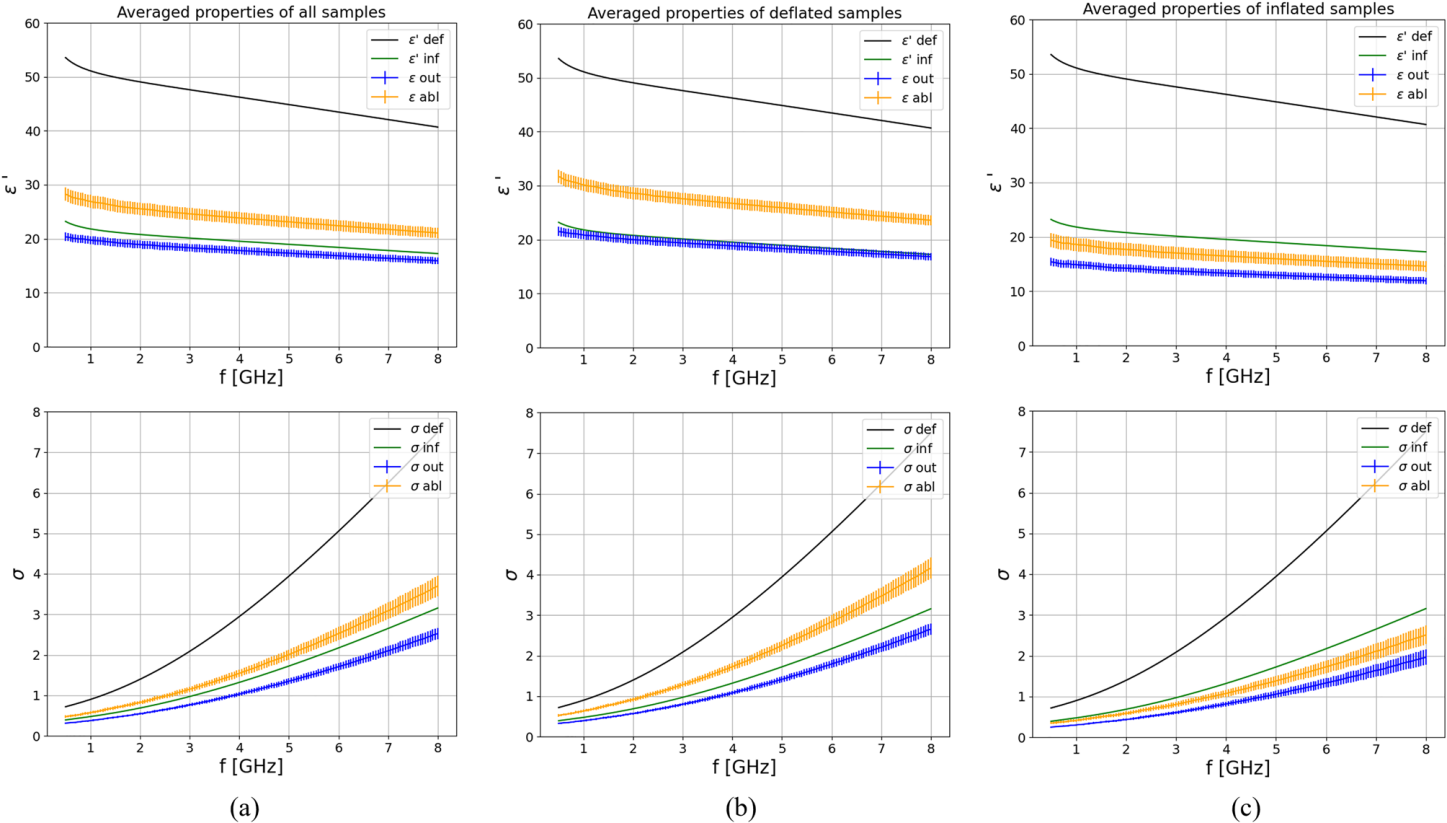
Comparison of dielectric properties of non-ablated (“out”) and ablated (“abl”) tissue, averaged across: (**a**) all samples regardless of the inflation state, (**b**) all deflated samples and (**c**) all inflated samples.

Further, dielectric properties of non-ablated and ablated lung were averaged across all samples regardless of the inflation level, and also pooled based on their deflated and inflated status. Figure 4 consists of three panels which show an overview of the dielectric properties of non-ablated and ablated tissue averaged across all samples [panel (a)], all deflated samples [panel (b)] and all inflates samples [panel (c)], respectively. The top row reports the relative permittivity, while the bottom row the equivalent conductivity. From the figure it can be noted that the dielectric properties of both non-ablated and ablated lung tissue are higher in case of deflated tissue in respect to the inflated one as previously reported by Vidjak et al.^19^. Additionally, in all subfigures the reference properties for inflated (labeled as “inf”) and deflated lung (labeled as “def”)^24,37^ tissue were plotted. When comparing the average measured properties of non-ablated inflated samples to those from literature [subfigure (c)], it can be noted that the obtained properties are very similar, which is not the case for non-ablated deflated samples [subfigure (b)]. The averaged deflated properties are significantly lower than the properties for deflated lung reported in literature^24^. This is because the deflated samples in this work are collapsed as received from the abattoir and still contained some air due to the alveolar structure of the tissue. However, as in the literature^37^, the properties of the inflated lungs are lower than those of the deflated lungs. In all cases, the increase of dielectric properties after ablation is sizable. The percentage increase of average ablated properties of all samples in respect to non-ablated ones is 38.4% for permittivity and 52.4% for conductivity. The percentage increase of all deflates samples is 41.9% for permittivity and 59.4% for conductivity, and that of inflated samples is 23.6% for permittivity and 32.2% for conductivity.

### 2-pole Cole & Cole model fitting

The non-ablated and ablated dielectric properties averaged across all samples were fitted to Eq. (9) using the W-LSM algorithm to minimize the error function. The fitting boundaries, used for both type of properties, are given in Table 2, and the obtained fitting parameters and the achieved fitting errors are presented in Table 3.

**Table 2:** Fitting boundaries for the 2-pole Cole–Cole model parameters fitting all averaged non-ablated and ablated lung tissue dielectric properties.

Boundary type	${\varepsilon }_{\infty }$	${\Delta }_{1}$	${\tau }_{1}$ (ps)	${\Delta }_{2}$	${\tau }_{2}$ (ns)	${\sigma }_{s}$
Non-ablated tissue						
Lower	2	3	6.5	10	29	0.001
Upper	4	35	231.1	1000	140	0.1
Ablated tissue						
Lower	1	3	6.5	20	10	0.01
Upper	5	35	12	900	120	0.2

**Table 3:** Obtained parameter values for 2-pole Cole–Cole model of all averaged non-ablated and ablated lung tissue dielectric properties.

Label	${\varepsilon }_{\infty }$	${\Delta }_{1}$	${\tau }_{1}$ (ps)	${\Delta }_{2}$	${\tau }_{2}$ (ns)	${\sigma }_{s}$	Fit. Er. ε′ (%)	Fit. Er. ε′′ (%)
Non.abl	2	16.71	6.5	505.01	29	0.08	1.25	1.07
Abl	1	24.50	6.5	397.57	10	0.001	1.22	0.96

It should be noted that although the frequency range of the measurements (0.5–8 GHz) does not include the exact frequencies at which the γ and β relaxation mechanisms occur, the influence of these two mechanisms is still visible in the considered range (Fig. 4). To this end, fitting the data to a single pole model (i.e. considering only γ relaxation) is not enough to accurately represent the behavior of the tissue at lower frequencies (i.e. below 1 GHz) because the tail of the β relaxation would be missing.

The results in Table 3 show a decrease in the ${\varepsilon }_{\infty }$ parameters for the ablated lung in respect to that of the non-ablated lung.

The parameter ${\Delta }_{1}$, connected to the γ relaxation mechanism, which is the relaxation of water, increased for ablated lung tissue. Such behavior is atypical for ablation as the tissue is losing water during the treatment and hence this parameter should decrease. In this case, it is possible that there is a tradeoff between the water loss and shrinkage of air cavities within the lung tissue, with the resulting density increase having a higher impact than the tissue water loss. In particular, the shrinkage of the air pockets in the lung cellular architecture causes the lung to become denser increasing tissue cellularity. In return, the dielectric probe’s sensing volume^38,39^ is filled with less air content when measuring post-ablation, and higher dielectric properties are measured. ${\tau }_{1}$ (ps), i.e., the relaxation time associated with γ relaxation (relaxation of the water), did not change with the tissue status although the tissue loses water. Such result can be explained that regardless of the water loss, the water remaining inside the tissue is still bounded in the same way.

The obtained values for β dispersion parameters ${\Delta }_{2}$ and ${\tau }_{2}$ (ns) decreased for the ablated lung in respect of the non-ablated tissue. The β dispersion is associated to passive cell membrane capacitance, intracellular organelle membranes and protein molecule response^34^. The tissue ablation results in cell necrosis, a state in which the cell organelles tend to swell, the cell membrane is ruptured and ultimately the cell lyses. Accordingly, a change in this parameter is expected^40^. When a decrease in the time constant ${\tau }_{2}$ is observed, a decrease in the characteristic capacitance and resistivity of the cell membrane can be assumed. Indeed, the characteristic capacitance is influenced by the permittivity of the cell membrane and its thickness^41^, altered during ablation by the cell rupture and lysis. More importantly, in case of cell membrane rupture more channels in the membrane are open and the cellular high-conductive materials are leaking causing the decrease in characteristic cell membrane resistivity^41,42^. In turn, these phenomena explain the decrease in the polarization time of β dispersion.

Finally, the, ${\sigma }_{s}$ parameter is linked to ionic currents and lower frequency mechanisms^43^. Considering that during ablation the tissue loses water, and in turn the ionic content, the decrease of this parameter is expected.

To evidence the role of air in the determination of dielectric properties of lung post ablation, the non-ablated and ablated dielectric properties were again fitted to Eq. (9) using the W-LSM algorithm considering separately the deflated and inflated samples. Table 4 gives the overview of the fitting boundaries used for the inflated and deflated non-ablated and ablated samples for W-LSM algorithm. The obtained parameter values of the Cole–Cole model, for either type of properties, are given in Table 5.

**Table 4:** Fitting boundaries for the 2-pole Cole–Cole model fitting of both non-ablated and ablated lung tissue dielectric properties for either inflated or deflated group.

Boundary type	${\varepsilon }_{\infty }$	${\Delta }_{1}$	${\tau }_{1}$ (ps)	${\Delta }_{2}$	${\tau }_{2}$ (ns)	${\sigma }_{s}$
Inflated properties						
Non-ablated tissue						
Lower	2	3	6.5	10	29	0.001
Upper	4	35	231.1	1000	140	0.1
Ablated tissue						
Lower	1	3	6.5	20	10	0.01
Upper	5	35	12	900	120	0.2
Deflated properties						
Non-ablated tissue						
Lower	1	3	6.5	30	25	0.01
Upper	4	35	231	900	120	0.2
Ablated tissue						
Lower	1	1	6.5	10	10	0.01
Upper	4	45	231	1000	140	0.2

**Table 5:** Obtained parameter values for 2-pole Cole–Cole model and fitting errors of non-ablated and ablated lung tissue dielectric properties for either inflated or deflated group.

Label	${\varepsilon }_{\infty }$	${\Delta }_{1}$	${\tau }_{1}$ (ps)	${\Delta }_{2}$	${\tau }_{2}$ (ns)	${\sigma }_{s}$	Fit. Er. ε′ (%)	Fit. Er. ε′′ (%)
Inflated non.abl	1.14	12.79	6.5	462.75	25	0.001	0.97	1.16
Inflated abl	1	16.20	6.5	283.87	10	0.001	1.16	1.18
Deflated non.abl	2.1	17.65	6.5	465.01	25	0.075	1.18	1.04
Deflated abl	1	27.56	6.5	434.72	10	0.01	1.21	0.99

The trends of the relaxation model parameters’ changes observed between the non-ablated and ablated tissue dielectric properties for both inflated and deflated groups are similar to those given in Table 3 for properties averaged across all samples. However, the increase of the ${\Delta }_{1}$ parameter post-ablation is quite small in the case of inflated lung. This result could be due to the effect of the continuous air supply to the tissue during the ablation and the following cooling process. The air pressure supplied may have limited the collapse of the air pockets in the ablated tissue.

### Histology validation of the ablated ovine lung tissue

Samples from the MWA TMD Lab group were analyzed to assess histological features linked to the ablation.

**Figure 5:**
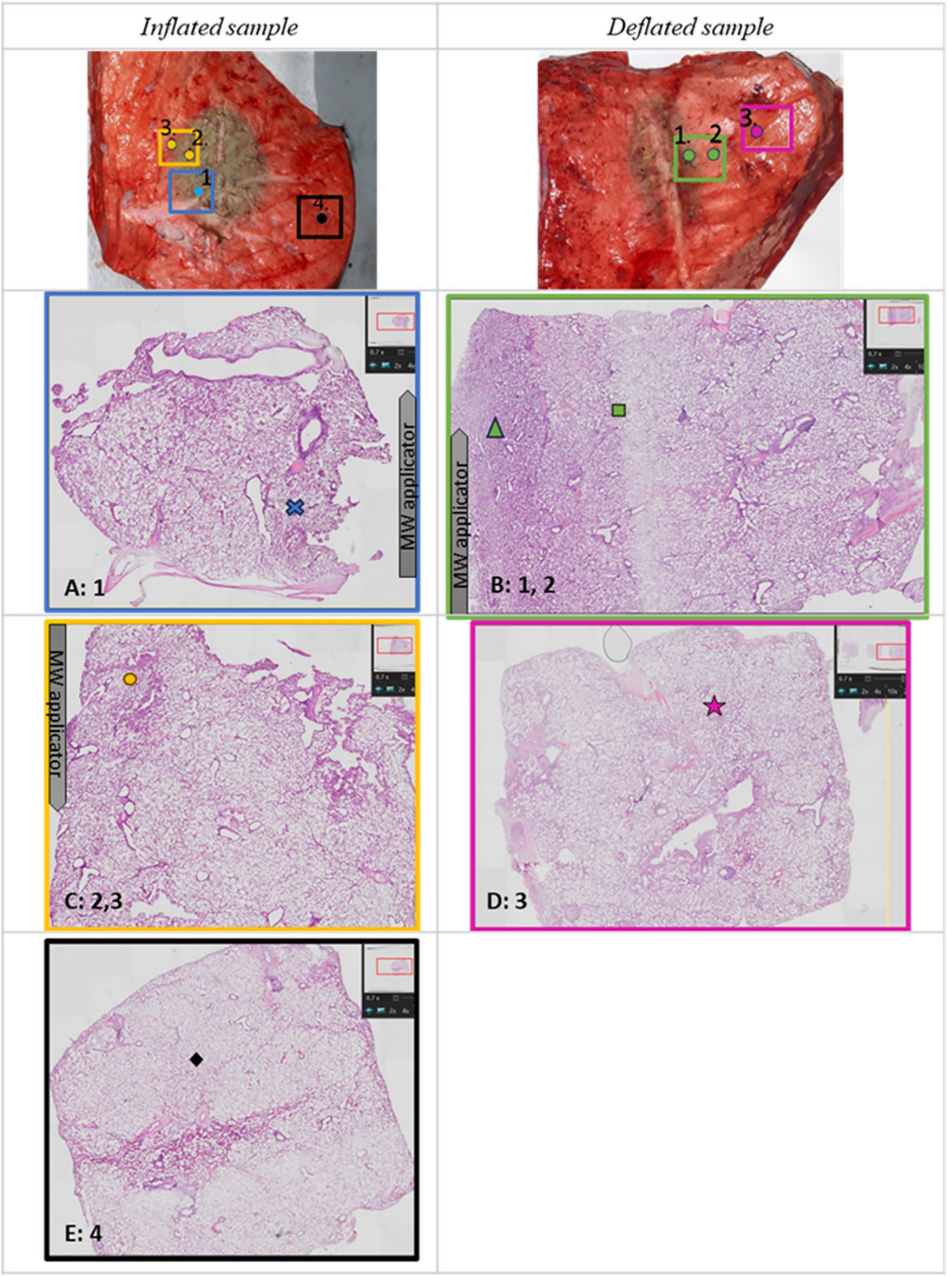
Comparison of gross and histology appearance of ablated and not ablated ovine lung tissue (the histological samples were obtained from the same ablated sample). An inflated sample (L6L2) is reported in the left column and a deflated sample (L4L2) in the right column. The top panels (**A,B**) show the gross image of the ablated sample. In these pictures, the areas sampled for histology, containing the measurement points, are marked and color coded. The histology appearance of those sampled areas are reported in (**C–G**) with ×0.7  magnification H&E: (**C**) contains the measurement point 1, (**E**) contains point 2 and 3, (**G**) point 4 of L6L2; (**B**) contains the measurement point 1 and 2, and paned (**D**) point 3 of L4L2. For the samples in contact with the antenna the position of the microwave (MW) applicator is shown. Then, in (**C–G**), symbols are used to mark specific areas reported magnified ×10  in Fig. 6.

Figure 5 reports an example of inflated (‘L6L2’) and deflated (‘L4L2’) lung tissue ablated in this study. The boxes marked on the gross sample images indicated the areas sampled for histological analysis containing the dielectric measurement locations (1, 2, 3 and 4 for ‘L6L2’ and 1, 2, 3 for ‘L4L2’). Panels from A to E report 0.7× magnified H&E images. The figures in panel A, C, E show the histological appearance of the samples collected from the ablated inflated sample ‘L6L2’; the figures in panel B, D show the histological appearance of the samples collected from ablated deflated sample ‘L4L2’. The following rows show the 2× magnified H&E images of each respective area.

The histology results allowed to identify three zones in the samples analyzed from the MWA TMD Lab:

1. An inner zone (zone i) of collapsed lung tissue, close to the antenna, with dense eosinophilic cytoplasm and condensed nuclei. In this zone air spaces are smaller due to shrinkage as a result of collapsed tissue. The cells have appearances of early ischaemic-type necrosis with dense cytoplasm of increased eosinophilia, and shrunken pyknotic nuclei. This zone corresponds to the innermost tan-brown area in the gross images.
2. A second zone (zone ii) of preserved alveolar architecture with relatively preserved alveolar space definition and with early signs of cytoplasm and nuclei alteration; at low power magnification this area had a pale appearance relative to the innermost zone. This area had appearances of increased cellularity of the interstitial tissue due to influx of inflammatory cells (macrophages) with some interstitial oedema.
3. A third area (zone iii) of preserved alveolar architecture without loss of alveolar space definition, nuclear chromatin, or alteration of the chromogenic qualities of the cytoplasm of cells. This area featured mild interstitial oedema and bordered peripheral normal lung tissue.

**Figure 6:**
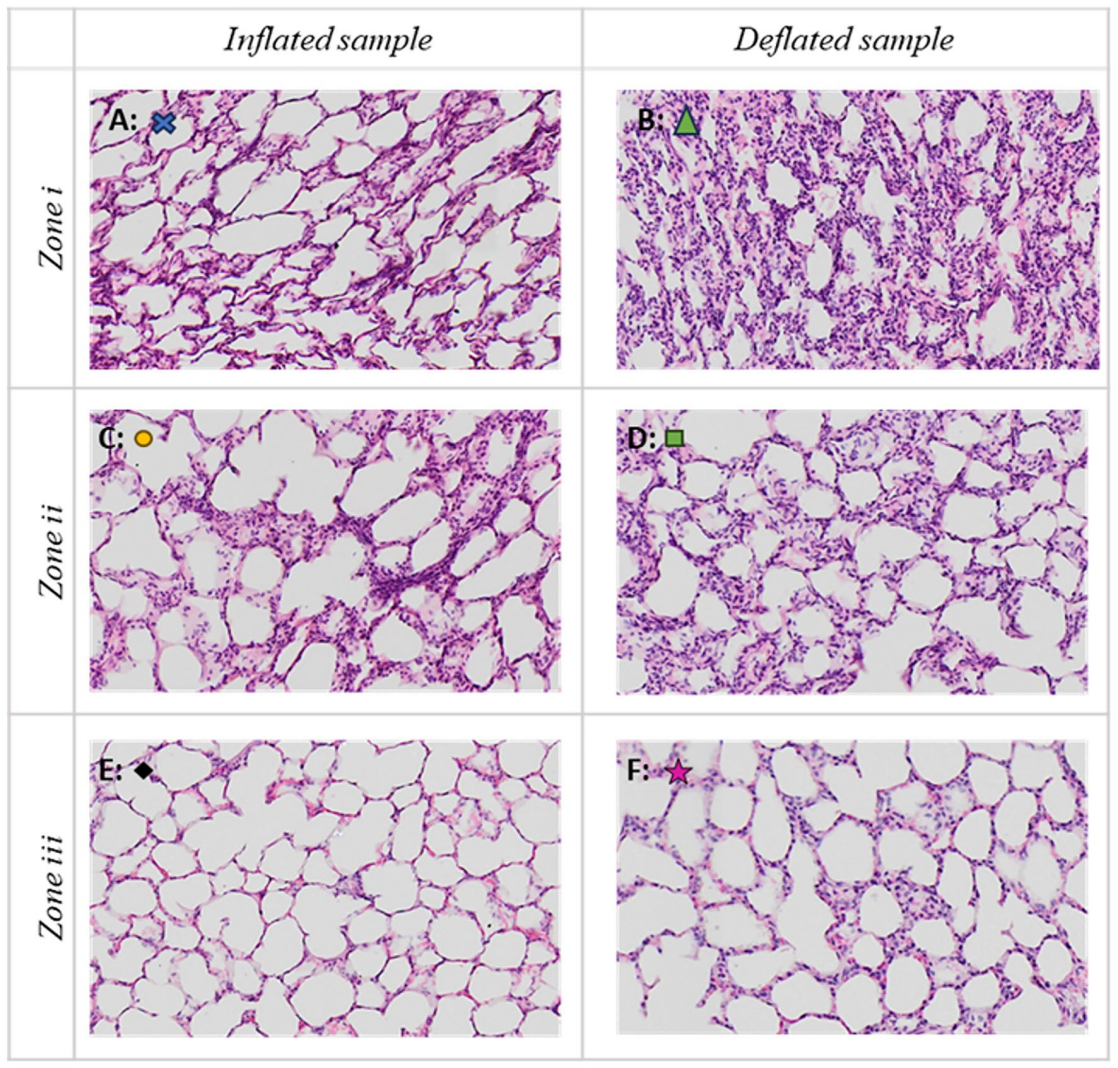
×10  magnification H&E images of the three areas identified by the histology analysis are reported: zone i with dense collapsed tissue (**A,B**), zone ii with less dense altered tissue with increased inflammatory cellularity and oedema (**C,D**) and zone iii of non-ablated tissue (**E,F**). (**A,C,E**) Obtained by an inflated sample (‘L6L2’), while (**B,D,F**) from a deflated sample (‘L4L2’). The symbols marks in the panels allow to locate the magnified areas in Fig. 5.

The three different zones identified and described are presented in Fig. 6. Figure 6 reports selected subareas of the sample of Fig. 5 magnified 10×. These subareas are marked with different symbols in Fig. 5, and presented in Fig. 6 with the corresponding symbols. Panel A and B are zone 1, C and D are zone 2, E and F are zone 3. Comparison of inflated (panels A, C and E) and non-inflated (panels B, D and F) tissue did not reveal any significant morphological differences.

### Microwave oven validation of the ablated lung tissue

**Figure 7:**
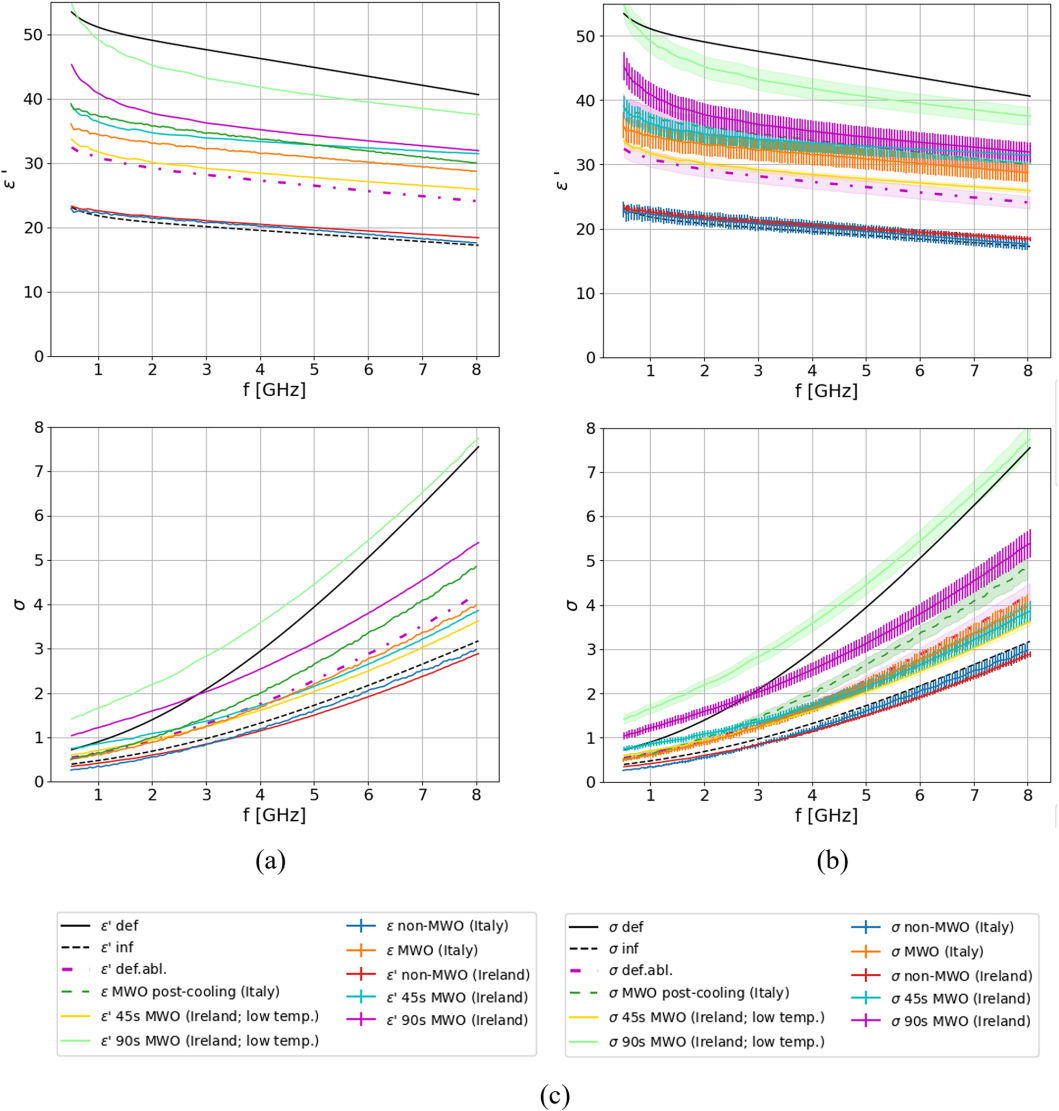
Averaged dielectric properties of samples measured in laboratories in Italy and Ireland: (**a**) without uncertainity; (**b**) with uncertainity; (**c**) legend.

Figure 7 shows the averaged dielectric properties of samples measured in both @TMD (“Ireland”) and @DIET (“Italy”) labs. The dielectric properties of the four ex-vivo ovine samples MWO heated at 800 W for 45 s and 90 s were averaged separately across all measurement locations and divided into two groups based on temperature. The data with higher temperature, recorded immediately after the MWO heating, are labeled as “45s MWO (Ireland)” and “90s MWO (Ireland)” respectively. The data with lower temperature, recorded after cooling, are labeled as “45s MWO (Ireland; low temp.)” and “90s MWO (Ireland; low temp.)” respectively. The dielectric properties of the five ex-vivo porcine samples heated at 120 W for 5 min were averaged across all samples and separated depending if the measurements were taken right after MWO heating [‘‘MWO (Italy)”], or after the 5-min cooling, [“MWO post-cooling (Italy)”]. “Healthy” labels refer to the pre-heating measurements, while “def” and “inf” report the literature data for deflated and inflated lung tissue respectively^24,37^. The curve labeled as “def.abl” represents the average dielectric properties of deflated ablated tissue defined with the 2-pole Cole–Cole model with parameters presented in Table 5.

The average of the measured temperatures of different measurement groups are given in Table 6. The average temperatures of measurements labeled as “45s MWO (Ireland)” and “45s MWO (Ireland; low temp.)” have a higher uncertainty then the rest of the measurements. This higher uncertainty originates from the fact that there were less measurement data; only two measurement locations were used immediately after the 45s after MWO heating, and only one location was used after the tissue cooled down. At each location, only three temperature measurement repetitions were performed (see Eq. (2)).

**Table 6:** Average temperatures and uncertainty of different measurement groups.

Measurement label	Average temperature (°C)
45 s MWO (Ireland)	$47.75\pm 4.21$
45 s MWO (Ireland; low temp.)	$32\pm 3$
90 s MWO (Ireland)	$44.89\pm 2.57$
90 s MWO (Ireland; low temp.)	$30.88\pm 0.87$
MWO (Italy)	$50.47\pm 0.94$
MWO post-cooling (Italy)	$36.72\pm 0.85$

From Fig. 7 it can be observed that the average dielectric properties, regardless of animal and laboratory, are very similar in case of non-heated tissue and aligns with the data reported in literature for lung parenchyma. Tissue MWO heated for 45 s [“45s MWO (Ireland)” and “45s MWO (Ireland; low temp.)”] and tissue MWO heated for 5 min at 120 W [“MWO (Italy)”] overlap within their experimental uncertainty, pointing to similar results given the same cumulative energy delivered (36 kJ). For the dielectric properties of deflated MTA heated tissue (“def. abl.”), ablated at 60 W 10 min (36 kJ), trend and values in conductivity similar to “45s MWO (Ireland)” and “MWO (Italy)” are observed, while slightly lower values are reported for relative permittivity of MTA ablated tissue for increasing frequencies. The amount of energy delivered to the tissue in the three above-discussed cases was the same in the specific timeframe. The longer time of the MTA treatment and the difference in the induced heat growth may have a role in the lower relative permittivity values reported. The tissue heated for additional 45 s [“90s MWO (Ireland)” and “90s MWO (Ireland; low temp.)] exhibits even higher properties caused by even greater tissue shrinkage which was evident immediately after the tissue was taken out of the oven. The tissue was very hard, dense, and carbonized at certain places. Additionally, the properties of samples MWO heated for 5 min increased further after cooling. The hypothesized reason for this behavior is that during this time, the physical processes in the tissue causing cell damage advances further.

**Figure 8:**
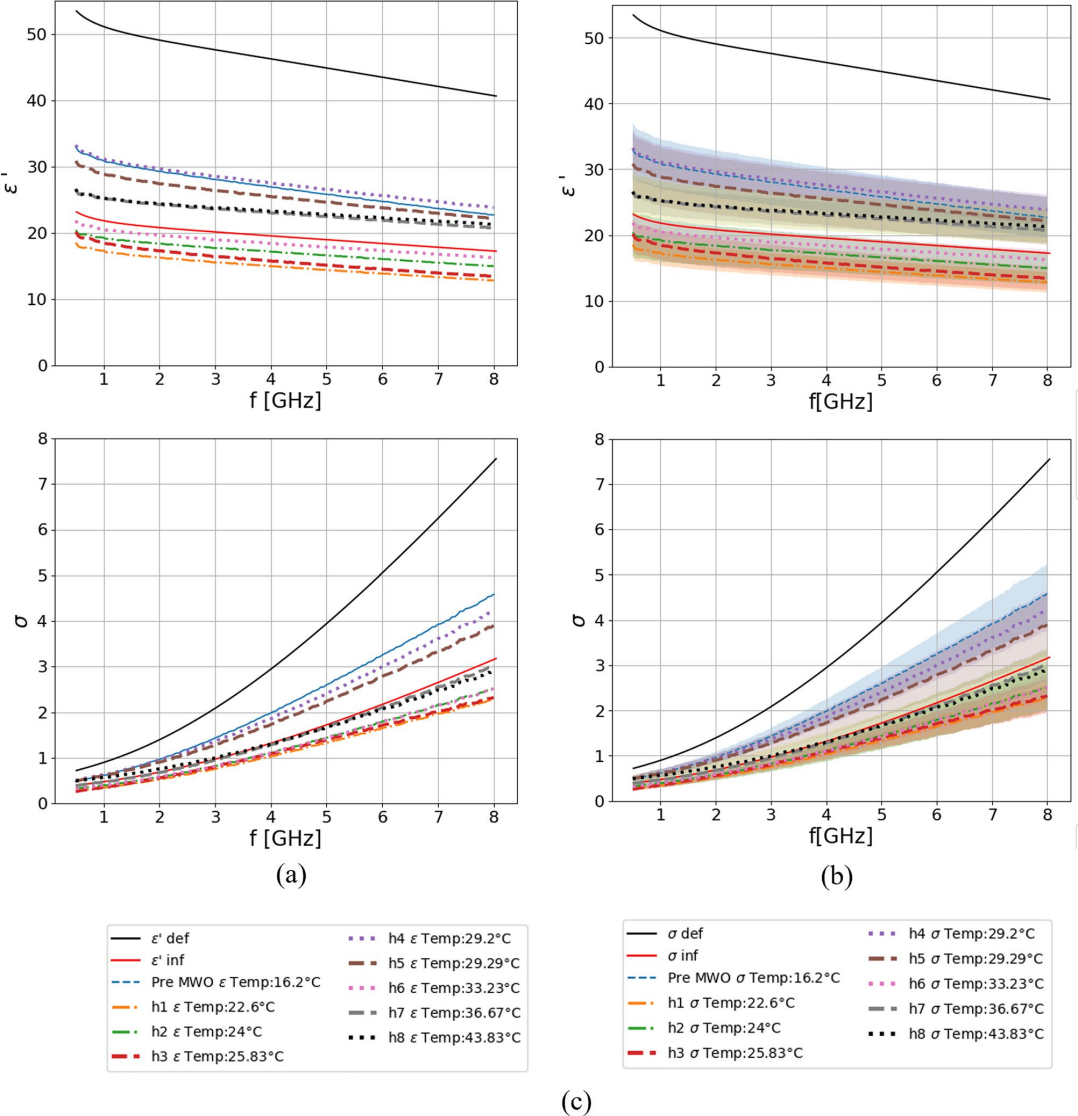
Measured dielectric properties of the porcine sample MWO heated several times for 45–90 s: (**a**) without uncertainty; (**b**) with uncertainty; (**c**) legend.; curves marked as ‘def’ and ‘inf’ represent properties of deflated and inflated lung measured at body temperature^24,37^.

Figure 8 shows the measured dielectric properties of the ex-vivo porcine lung sample which were MWO heated several times for 45–90 s aiming to obtain a 5 °C temperature increase after each heating session. The curves of dielectric properties are obtained by averaging 3–6 measurement repetitions depending on the heating session and they are labeled with “hx” where “x” represents the order number of the heating session. Additionally, each label contains the average value of the temperature measured during the measurement of dielectric properties. The dielectric properties of tissue measured before the start of the experiment are also present and labeled as “pre MWO”. Additionally, the curves of deflated and inflated lung reported by Gabriel et al.^24^ and measured at body temperature are labeled in the figure as “def” and “inf”, respectively.

It can be observed that dielectric properties of lung tissue, in this experimental setup, show no monotonical behavior with the increasing temperature of the different MWO sessions. In particular, at some time point they decreased with increasing temperature (h1, h3, h5, h6), while in others the properties increased with increasing temperature (h2, h4, h7). Finally, in the final step (h8) they remained almost identical to those measured in the previous one (h7). This behavior could be connected to the temperature instability of the sample once it is taken out from the oven. The hypothesis behind the drop in dielectric properties after MWO heating which lasts only 45 s at 120 W is that this process dehydrates, rather than ablates the tissue, i.e. the tissue loses water content, but its structure remains the same. Although, the time duration is too short to fully ablate/carbonize the tissue, in some cases the properties after a certain MWO session increase in respect to the previous one (i.e. ‘h4’ in respect to ‘h3’ shows an increase in dielectric properties). Such behavior can potentially be caused by the water content from deeper parts of the tissue being pushed towards the surface.

## Discussion and conclusions

In this study, the dielectric properties of ex-vivo animal lung tissue post-MTA treatment have been investigated. Thirteen ex-vivo ovine lung samples were characterized before and after 10-min ablations at 2.45 GHz and 60 W. The measured properties were fitted to 2-pole Cole–Cole models. The analysis showed that unlike other results reported in the literature, the dielectric properties of lung tissue tend to increase after ablation^21^. This is likely due to lower heat modalities used in other works. Furthermore, they were measuring the dielectric properties in correlation to the temperature that they achieved by slowly heating the sample, and they did not perform measurements before and after the ablation treatment once the sample has cooled down back to the room temperature. Considering that lung is a sponge-like, air-dominated tissue, the plausible cause for this behavior is the shrinkage of air cavities forming the structure of this tissue due to the release of great heat during ablation. This hypothesis was confirmed with histological analysis of some of the ablated samples. In the ablated area, two zones can be identified, one denser and the other one less dense; in Sebek et al. 2020^31^, in vivo, a similar histological pattern was observed. They described inner zones (1 and 2) of tissue post-ablation with features similar to those described here, however a greater delineation of the features was observed in porcine lung ablated in vivo with 5 distinct areas of pathological change reported. The areas close to the position of the ablation antenna exhibited a denser configuration of tissue in respect to those further away, in the untreated section of the tissue. When observing the obtained Cole–Cole model parameters for non-ablated and ablated samples, particular changes were observed and explained in the context of physical changes experienced by the tissue during the ablation. Parameters ${\Delta }_{1}$ and ${\uptau }_{1}$ are parameters connected to the γ-dispersion (relaxation of bounded water in the tissue): ${\Delta }_{1}$ increases although the tissue loses water, and this can be explained by the fact that there is a tradeoff between the water loss and air cavity shrinkage; ${\uptau }_{1}$ remains the same, and this is probably because the remaining water remains bounded in the same way. Parameters ${\Delta }_{2}$ and ${\uptau }_{2}$ are related to the β-dispersion (relaxation of passive cell membrane capacitance, intracellular organelle membranes and protein molecule response): ${\Delta }_{2}$ and ${\uptau }_{2}$ decrease which can be explained by the cell-membrane rupture. Finally, ${\sigma }_{s}$ also decreases and it is due to the water loss, and consequently, loss of ionic currents.

To verify the results obtained when the tissue is heated with MTA applicators, five ex-vivo porcine samples were heated in MWO for 5 min at 120 W to match the energy deployed into the tissue during the ablation. The obtained dielectric properties post ablation and post MWO-heating were very similar. Furthermore, four ex-vivo ovine samples were heated twice in the MWO for 45 s at 800 W. The first MWO session in this case matched the energy of the MTA procedure and the measured properties were also very similar, while the properties measured after the second MWO session showed an even further increase in properties. The obtained dielectric properties of the non-ablated in both 5-min at 120 W and 45 s at 800 W were almost the same and the properties measured immediately after the MWO-heating were within the measurement uncertainty of each type of experiment.

Finally, 1 ex-vivo porcine sample was used to re-create a slow-heating MWO experimental setup from literature. This experiment aimed at increasing the temperature of the sample for 5 °C after every 45 s in the oven at 120 W. This showed to be unfeasible, and the sample had to remain inside the MWO more than 45 s to achieve such temperature increase. The measured dielectric properties after each MWO session were very variable, but in 4 of 8 cases the properties decreased with the increase in temperature. This indicates that the change in dielectric properties of lung samples is directly linked to the heating rate of the sample. Slow MWO heating resembles more a dehydration process and the dominant mechanism occurring in the tissue is water loss. Fast MWO heating and MTA deploy a large amount energy in the tissue and there is both water loss and tissue shrinkage occur. This hypothesis was also validated by histological analysis where a deformation of air cavities is observed in the ablation zone, and it becomes more prominent in the areas closer to the position of the MTA antenna.

From this analysis it is evident that there are many factors potentially influencing the dielectric properties of lung tissue, such as inflation level, temperature and heat-deployment rate. The findings in this study give a better insight into the changes occurring in lung tissue post-ablation and can potentially allow recovery process predictions and more accurate MTA applicator designs. The future studies need to focus on finding an adequate technique for reliable in-situ measurement of dielectric properties of lung tissue during the ablation and in relation to temperature.
